# A rare case of dysplasia epiphysealis hemimelica in a hip joint treated by arthroscopic tumour resection: a case report

**DOI:** 10.1093/jhps/hnaf064

**Published:** 2025-10-21

**Authors:** Kosuke Sumi, Naomi Kobayashi, Yohei Yukizawa, Shota Higashihira, Masafumi Naito, Ken Kumagai, Hyonmin Choe, Yutaka Inaba

**Affiliations:** Department of Orthopaedic Surgery, Yokohama City University Medical Center, 4-57 Urafune-cho, Minami-ku, Yokohama, Kanagawa 232-0024, Japan; Department of Orthopaedic Surgery, Yokohama City University Medical Center, 4-57 Urafune-cho, Minami-ku, Yokohama, Kanagawa 232-0024, Japan; Department of Orthopaedic Surgery, Yokohama City University Medical Center, 4-57 Urafune-cho, Minami-ku, Yokohama, Kanagawa 232-0024, Japan; Department of Orthopaedic Surgery, Yokohama City University Medical Center, 4-57 Urafune-cho, Minami-ku, Yokohama, Kanagawa 232-0024, Japan; Department of Orthopaedic Surgery, Yokohama City University Medical Center, 4-57 Urafune-cho, Minami-ku, Yokohama, Kanagawa 232-0024, Japan; Department of Orthopaedic Surgery, Yokohama City University Medical Center, 4-57 Urafune-cho, Minami-ku, Yokohama, Kanagawa 232-0024, Japan; Department of Orthopaedic Surgery, Yokohama City University, 3-9 Fukuura, Kanazawa-ku, Yokohama, Kanagawa 236-0004, Japan; Department of Orthopaedic Surgery, Yokohama City University, 3-9 Fukuura, Kanazawa-ku, Yokohama, Kanagawa 236-0004, Japan

## Abstract

We report a rare case of dysplasia epiphysealis hemimelica (DEH) in a hip joint treated by arthroscopic tumour resection. A 12-year-old male developed difficulty walking due to progressive right hip pain that had persisted for 3 months. Radiography revealed subluxation of the right hip, and magnetic resonance imaging revealed a prominent elevated lesion on the acetabular roof. DEH of the hip was suspected; the tumour was resected *via* arthroscopy, which resolved the hip pain and subluxation. This is the first case report of DEH originating from the acetabulum fossa that was treated using arthroscopic tumour resection, presenting an effective and minimally-invasive treatment option.

## Introduction

Dysplasia epiphysealis hemimelica (DEH), also known as Trevor disease, is an extremely rare benign bone tumour [1] characterized by single or multiple excessive growths of osseocartilaginous tissue on one side of the epiphysis. DEH most frequently occurs in the lower extremities, especially the tarsal bones, distal tibia, and distal femur [2]. The ankle and knee joints are most commonly affected; it has been reported to occur in the hip joint in only a few patients [3, 4]. Despite its benign nature, progressive symptoms, such as pain and restricted range of motion, are characteristic as the mass increases in size. As growth progresses, hyperplasia advances, leading to increased mechanical stress and joint incongruity [5], ultimately resulting in irreversible joint damage [6]. This is particularly concerning in the hip joint, where these changes and the resulting subluxation can considerably limit daily activities. Early diagnosis and intervention are essential for preserving joint function and preventing long-term disabilities.

In most previously-reported cases, treatment had involved tumour resection accompanied by surgical dislocation. However, surgical dislocation carries concerns with greater invasiveness and postoperative complications, such as femoral head necrosis and osteoarthritis progression. We report a case in which we performed arthroscopic tumour resection of the hip joint and achieved good clinical outcomes. Written informed consent for the publication of these data was obtained from the patient and his parents.

## Case report

A 12-year-old boy was referred to our hospital for evaluation and treatment of right hip pain that had persisted for three months. The patient had developed Kawasaki disease during infancy but achieved remission and had no other relevant medical history. He experienced right hip pain under load and had an unsteady gait, resulting in the use of crutches. His hip joint range of motion was limited to 65° of flexion, 15° of abduction, and 0° of extension, with a Modified Harris Hip Score of 59.4 points. Blood tests indicated no obvious abnormalities or signs of inflammation. Plain radiography revealed right hip subluxation (Fig. 1). Plain magnetic resonance imaging of the hip joint revealed a protruding lesion continuous with the ilium at the acetabular floor, which showed high signal intensity on T2-weighted imaging (Fig. 2A and B). Based on physical and imaging findings, we suspected DEH and decided that it was possible to approach the lesion and perform arthroscopic resection of the hip joint.

**Figure 1:**
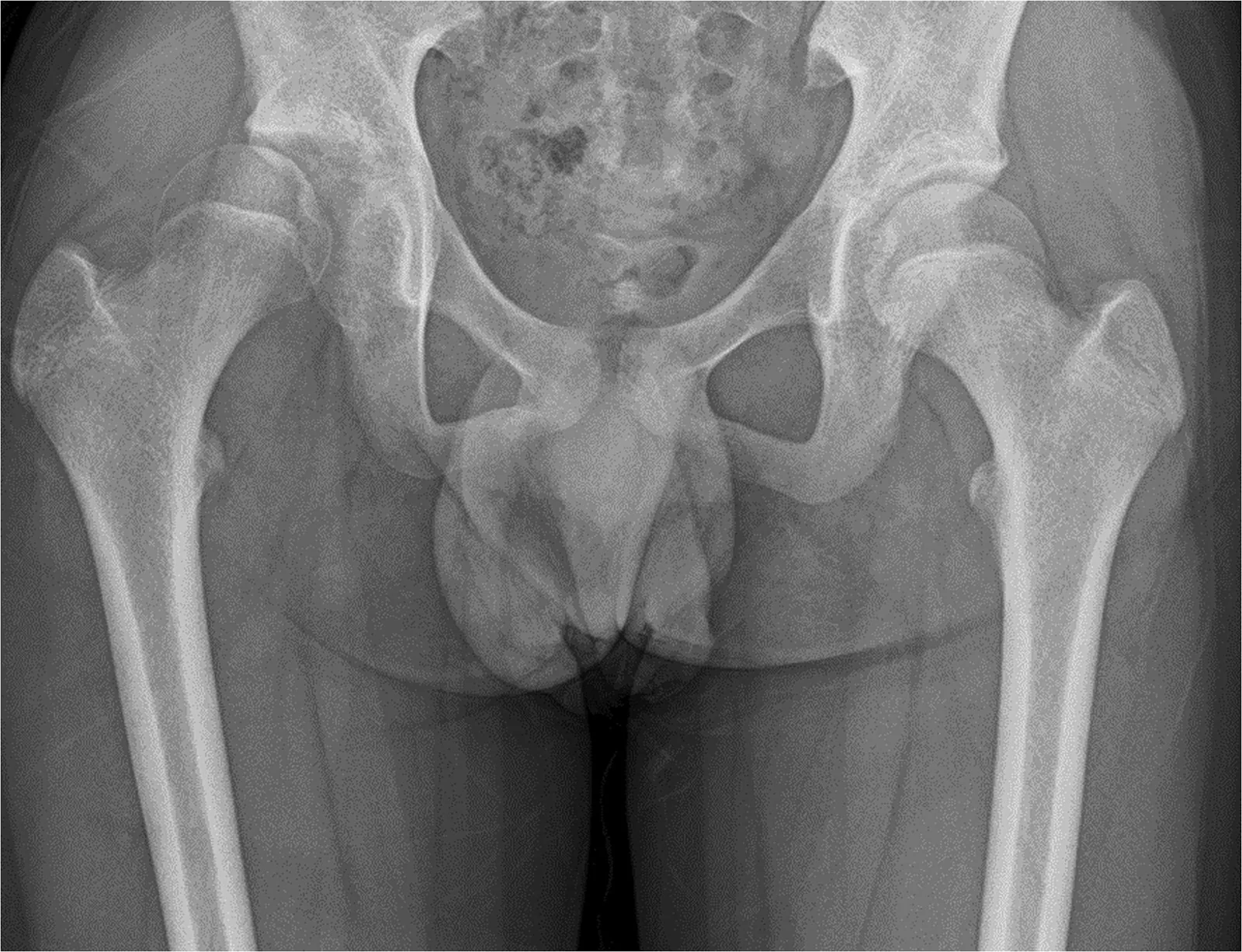
Plain radiography revealing subluxation of the right hip at initial consultation.

**Figure 2:**
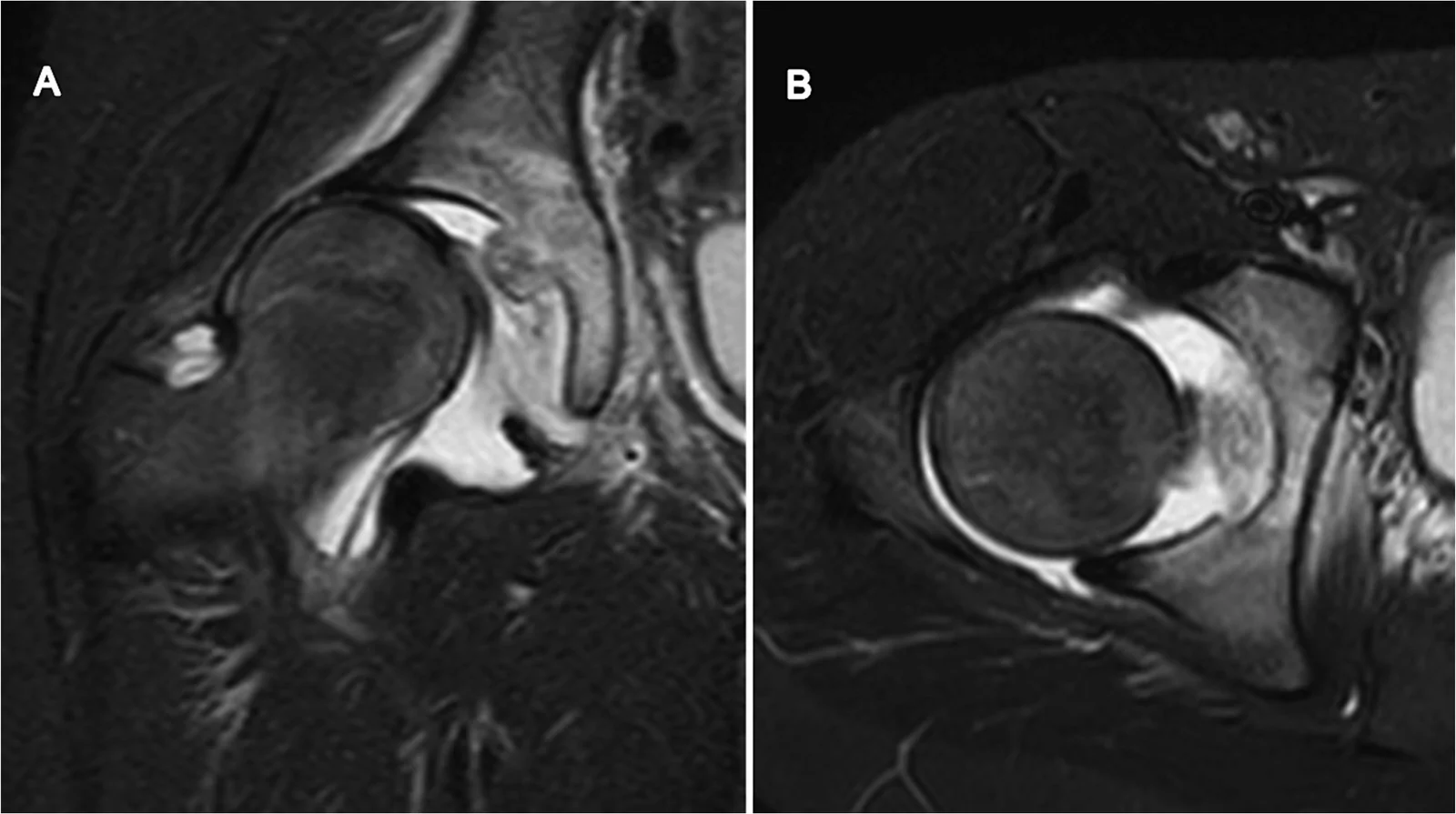
Plain magnetic resonance imaging of the hip joint revealed a protruding lesion continuous with the ilium at the acetabular floor, (A) coronal and (B) axial views of T2-weighted plane magnetic resonance imaging at initial consultation.

Arthroscopic observation of the hip joint was performed using the anterolateral and mid-anterior portals with traction, revealing synovial proliferation and a protruding lesion on the acetabular floor (Fig. 3A). Because it was difficult to remove the lesion as a single mass, it was excised piecewise (Fig. 3B and C). After tumour removal, the cancellous bone on the acetabular floor was exposed (Fig. 3D). Pathological tissue examination revealed osteocartilaginous tissue with a thick, irregular cartilage cap and noncontiguous ossification centres (Fig. 4), consistent with previous reports [7, 8].

**Figure 3:**
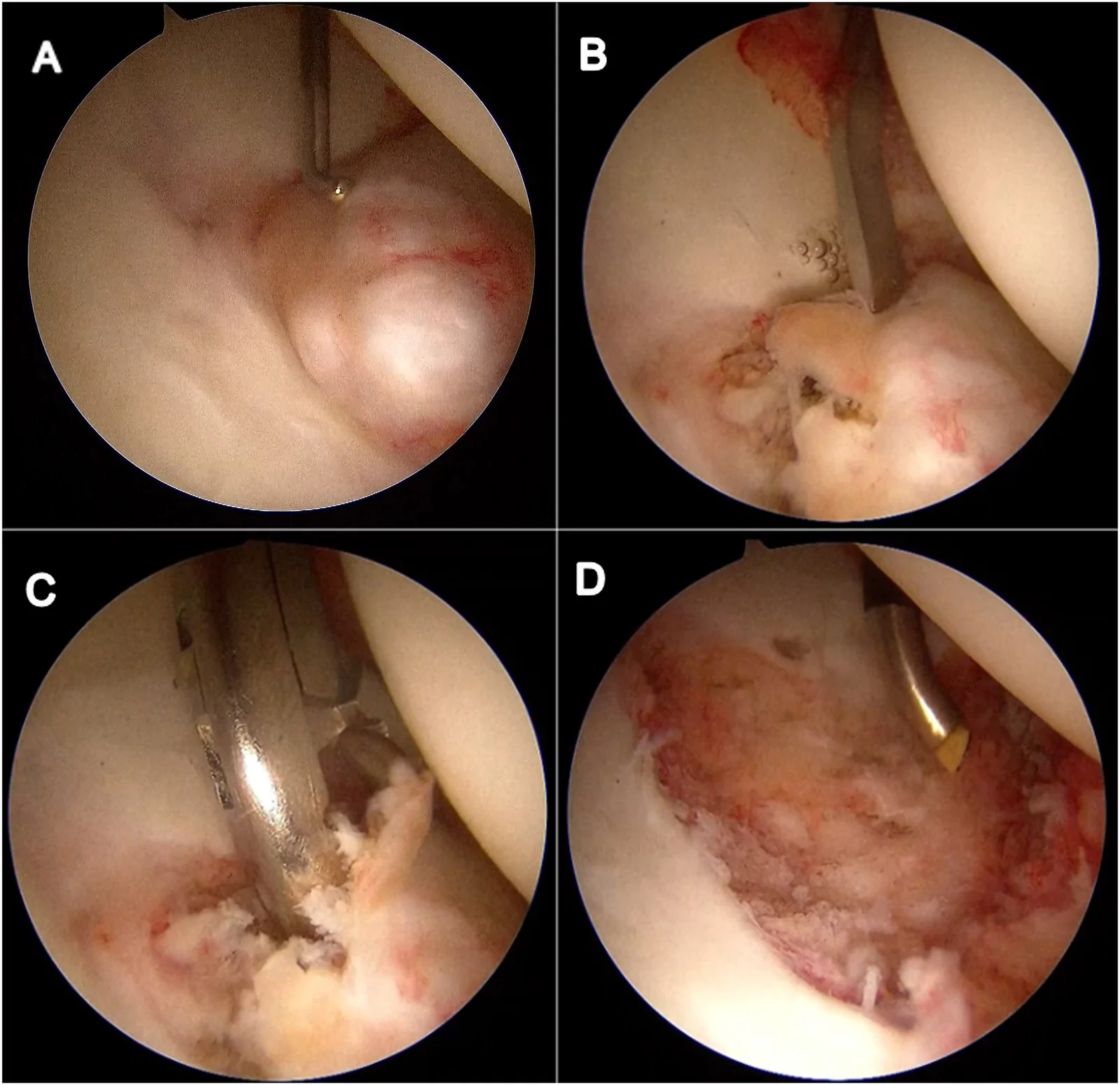
Arthroscopic observation of the hip joint was performed using the anterolateral and mid-anterior portals with traction, revealing synovial proliferation, (A) a protruding lesion on the acetabular floor is observed arthroscopically. (B, C) The protruding lesion is excised piece by piece. (D) Following the excision of the tumour, the cancellous bone becomes visible on the acetabular floor.

**Figure 4:**
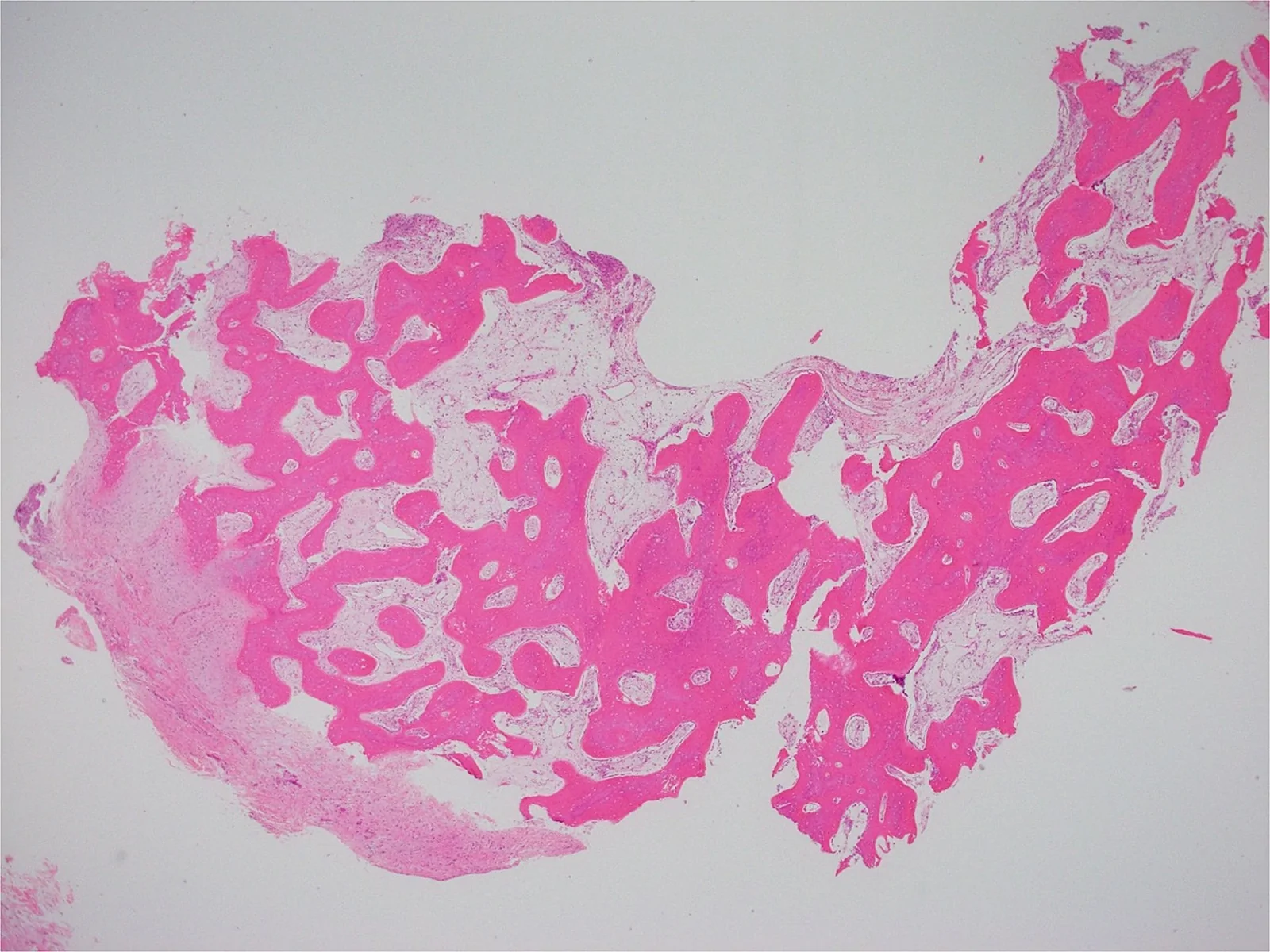
Pathological tissue examination revealing osteocartilaginous tissue with a thick irregular cartilage cap and non-contiguous ossification centres (original magnification: ×100).

Improved joint congruency and maintenance of the centripetal position were confirmed postoperatively using plain radiography (Fig. 5). After 2 week of non-weight bearing and 2 week of half-weight bearing, the patient began full-weight bearing 4-week postoperatively. Jogging was permitted 3 months after surgery, and the patient eventually returned to sports. The Modified Harris Hip Score improved to 95.7 points 3 months after surgery and was maintained at a perfect score of 100 from 6 months postoperatively through to the 2-y final follow-up. His hip joint range of motion improved to 120° flexion and 40° abduction at the final follow-up.

**Figure 5:**
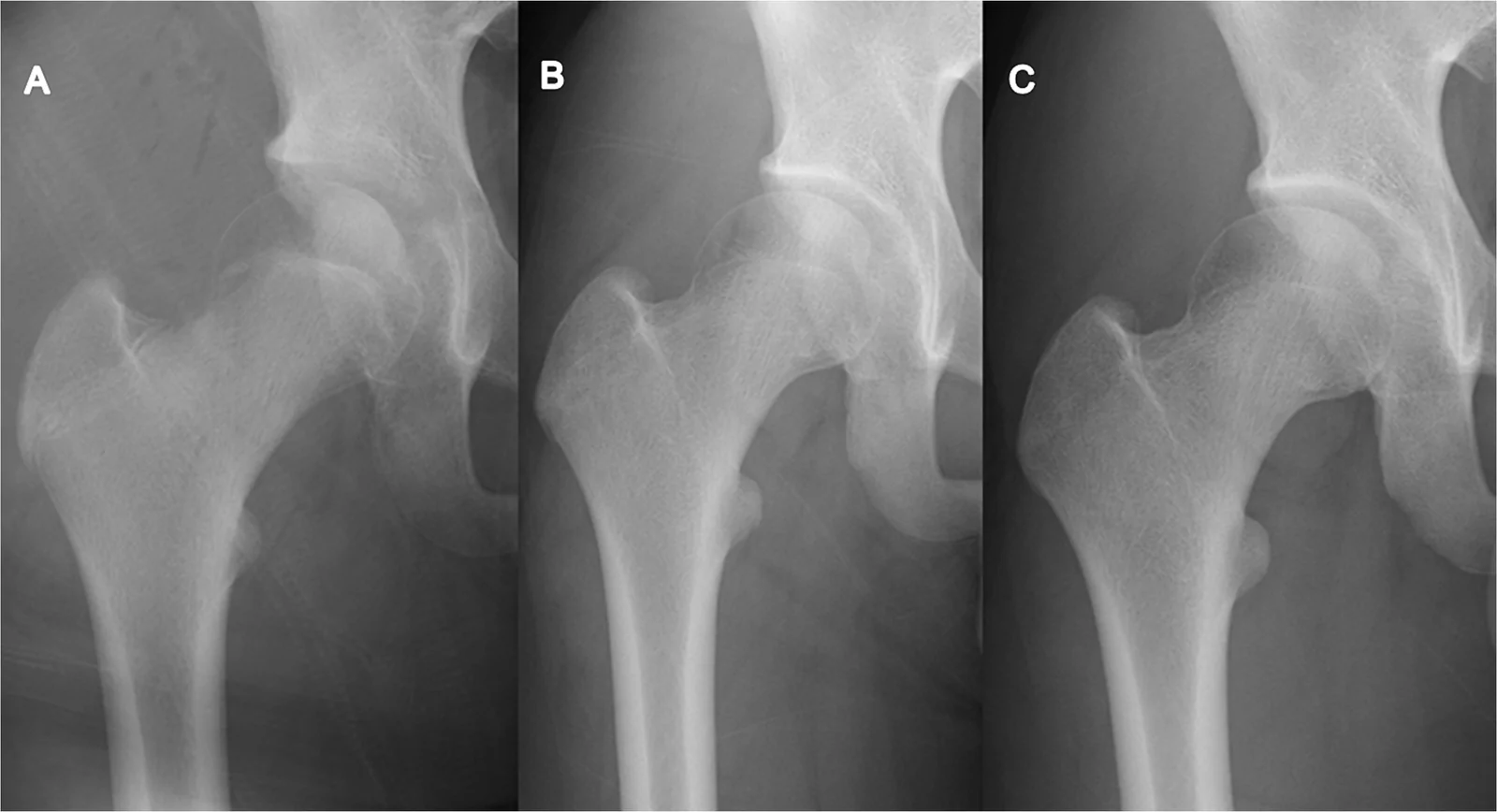
Joint congruity has been maintained for 2 years following surgery. (A) Day of surgery, (B) 1-year postoperatively, and (C) 2-years postoperatively.

## Discussion

We performed arthroscopic tumour resection in a 12-year-old male who presented with progressive hip pain and limping for 3 months, with favourable short-term postoperative outcomes. To the best of our knowledge, this is the first case report of DEH in a hip joint treated with arthroscopic tumour resection. Intra-articular DEH of the hip causes joint incongruity, which can lead to subluxation and functional impairment if it progresses. Confirmation of tumour lesions and pathological examination are essential for a definitive diagnosis.

Ten previous case reports of DEH in the acetabulum exist. Tumour resection with surgical dislocation was performed in eight cases [3, 4, 9–12], and total hip arthroplasty was carried out in one case [6]. In a case complicated by developmental dysplasia of the hip, triple pelvic osteotomy was also performed [13]. In five cases, tumour lesions were observed not only in the acetabulum but also in the proximal femoral epiphysis and varus derotation osteotomy was performed following tumour resection in two cases [9, 10]. The greatest advantage of hip arthroscopy is that it allows access to lesions without causing dislocation. In particular, when a definitive diagnosis cannot be made pre-operatively, arthroscopic surgery can be an effective strategy which allows for thorough observation and tissue sampling. Surgical treatment involving surgical dislocation is one option; however, surgical dislocation necessitates greater invasiveness and carries the risk of postoperative femoral head necrosis.

Hip arthroscopy, which enables a minimally-invasive assessment of the joint, collection of tissue specimens, and treatment, is a useful therapeutic option; however, careful consideration must be given to surgical indications regarding tumour location and size. Hip arthroscopy is a technically demanding procedure because of the deep location of the hip joint and its complex anatomy. The posteromedial corner of the central compartment has been consistently identified as the most difficult area to access with standard arthroscopic techniques [14]. Traction is essential for full access to the acetabular fossa [15]. Arthroscopy is generally preferred for small, well-defined, and benign tumours; large, malignant, or poorly-accessible tumours may require open surgery for complete and safe removal. Prior assessment of lesion distribution and tumour size through imaging studies is essential, and it is necessary to select appropriate candidates. Further studies are required regarding these indications.

In conclusion, a rare case of DEH in acetabular fossa was presented, which was treated successfully by arthroscopic tumour resection. Thus, arthroscopic tumour resection is an option for the treatment of hip joint DEH.

## Data Availability

The data underlying this article will be shared on reasonable request to the corresponding author.
